# Evaluation of the psychometric properties of the Greek version of the Active Life with Asthma (Gr-ALMA) review: a descriptive methodological study

**DOI:** 10.1186/s12913-019-4155-5

**Published:** 2019-05-22

**Authors:** Chris Livadiotis, Ekaterini Lambrinou, Vasilios Raftopoulos, Nicos Middleton

**Affiliations:** 1 Department of Nursing, School of Health Sciences, Cyprus University of Technology, Limassol, Cyprus; 2 Emergency Department, Nicosia General Hospital, Nicosia, Cyprus; 3 Hellenic Centre of Disease Control and Prevention, Athens, Greece

**Keywords:** Asthma, Self-management, Asthma control, Metric properties, Quality of life

## Abstract

**Background:**

Regular asthma reviews are recommended by international guidelines to improve the quality of life of asthma patients. To facilitate these reviews in primary care practice, there is a need for structured asthma review tools.

**Aim:**

The aim of this study was to assess the metric properties of the Greek-translated version of the Active Life with Asthma (ALMA) review.

**Methods:**

A convenience sample of 156 asthmatic patients from three public hospitals participated in this methodological study with a descriptive cross-sectional correlation design. Participants responded to the 19-item ALMA questionnaire and provided socio-demographic and clinical information. The construct validity of the tool was explored in exploratory factor analysis and the internal consistency of scale and sub-scales was estimated using Cronbach’s α. Convergence validity was assessed using the Asthma Control Questionnaire (ACQ), a commonly used asthma control measure, and concurrent criterion validity was assessed using the MiniAQoL, an asthma-specific quality of life questionnaire. Known-group validity was assessed based on observed differences in terms of frequency of hospitalizations or emergency visits in the past year.

**Results:**

Amongst 156 participants, 95 (60.9%) were women and the median age was 50–65 years old. Exploratory factor analysis (KMO = 0.83 and Bartlett test < 0.001) with principal component extraction and orthogonal rotation revealed a clear structure of three factors with little cross-loading: physical, environmental and mental domains, as in the original study. Cronbach’s alpha coefficient for internal consistency for the whole scale was 0.85, while for the sub-scales, these were: environmental a = 0.69, mental a = 0.76 and physical a = 0.85. Test-retest reliability based on the correlation between scores of 20 participants responding twice two weeks apart was r = 0.92. There was stong correlation in the expected direction between ALMA and ACQ (r = − 0.70) as well as miniAQoL (r = 0.71). Finally, there were statistically significant higher ALMA scores in participants who reported emergency visits and hospital admissions in the past year.

**Conclusion:**

In general, the ALMA showed good metric properties. It appears to be a reliable and valid tool which can be used as a measure for asthma control and self-management in clinical practice as well as future descriptive or intervention research studies.

## Introduction

Asthma is considered a major public health issue. Globally, the prevalence of asthma is rising [14]. Symptom control is the primary goal of treatment. Nevertheless, there is evidence to suggest that asthma is frequently underdiagnosed and undertreated. In addition, among people diagnosed with asthma, the extent to which they have an active role in the day-to-day management of their condition is directly associated with improved outcomes [14]. Self-management and long-term control of mild to moderate asthma is often poor and this has been linked to several factors, including the reluctance to accept diagnosis of a chronic condition, the complexity of the condition, over-reliance on health professionals or simply forgetfulness [14].

It has been suggested that the financial costs associated with asthma care continue to increase, yet improved care with a focus on better self-management remains suboptimal. Promoting optimal self-management, including the use of asthma action plans along with regular health professional reviews, has been shown to be an effective strategy and is recommended by asthma guidelines internationally [16].

Studies from several countries (USA, UK, Sweden) have shown the importance of self-management in all chronic diseases and especially for asthmatics in improving knowledge and quality of life [12]. Asthma control depends on patients acquiring information and developing self-management skills to be applied in the long term [11]. It has been shown that self-management lowers the burden of illness as perceived by patients with asthma and is a safe basis for intermittent treatment with inhaled corticosteroids [18].

The Active Life with Asthma – ALMA, is a relatively new tool developed and tested in Sweden to facilitate structured review and management of asthmatic patients in primary care [10]. Unlike previous tools, the ALMA taps on other aspects of asthma control and self-management such as the mental domain or environmental asthma triggers (e.g. exposure to smoke, dust or pollen). The authors concluded that, to quote: “The breadth of the questions in the ALMA tool and the pragmatic use in clinical practice suggest that it can form the basis of a structured review in primary care which may translate into improved outcomes”.

The purpose of this study was to assess the metric properties of the Greek-translated version of the Active Life with Asthma (ALMA) review among a sample of Cypriot adult asthma patients. Specifically, the construct, convergence (against ACQ), concurrent criterion (against a quality of life measure) and know-group validity (against asthma severity indicators) validity were examined. As far as we are aware, this is the first study to use and test the metric properties of the ALMA questionnaire internationally.

## Methods

### Study design and sample

A methodological study with a descriptive cross-sectional correlational design was performed. Eligible participants were patients with doctor-diagnosed asthma over 18 years old, either attending the pulmonary clinics of three public hospitals (Nicosia, Larnaca, Limassol) as part of their regular consultation visit or who were hospitalized at the pulmonary wards of these hospitals during the study period. Excluded were patients under 18 years of age arriving at the emergency department or those who did not wish to participate. The sample was a convenience consecutive sample of asthma patients in the recruitment period until a minimum required sample size was achieved. The study aimed for 160 completed questionnaires in order to permit the exploration into the dimensionality of the tool based on the participants’ responses (i.e. 10 participants per item of the scale-part of the tool).

### Measurement tools

The instruments used in the study are (1) ALMA, (2) ACQ and (3) miniAQoL. Permission to use all tools was obtained by the developers. In addition, general sociodemographic and clinical characteristics were also collected in order to assess potential differences in asthma control and quality of life across sub-groups. Specifically, these included gender, age, smoking status (pack-years), educational attainment, self-reported medication use, allergies, comorbidity and family history.

The Active Life with Asthma (ALMA) tool was developed with the purpose to provide a tool to structure asthma review consultations in primary or secondary care settings. The developmental process of the tool is described in detail by Kiotseridis et al. [10]. In brief, it involved focus groups with a diverse group of asthma patients recruited from primary care settings in Sweden with the aim of identifying unmet needs. The initial version of the questionnaire contained 25 questions and was later reduced to 19, of which a total of 16 questions refer to the level of control of asthma symptoms and form the scale-part of the tool. Each of these items have a four-point response scale (often, sometimes, seldom, never). An additional 2 questions with yes/no answers and 1 question about as-needed medication are included in the questionnaire. The paper-form questionnaire was initially validated against the 5-item Asthma Control Questionnaire (ACQ) in a postal survey of 200 consecutive patients with a doctor diagnosis of asthma. It was later introduced as an optional web-based application in a number of primary care centres and underwent further validation among a larger sample of over 1700 asthma patients.

The Asthma Control Questionnaire (ACQ) includes the five main clinical symptoms: night-time waking, symptoms on waking, activity limitations, shortness of breath and wheezing and one question about b2-agonist use [7]. In its 7-item form, it normally also includes FEV1, which was nevertheless not used in the current study as it requires spirometry. Patients respond to each question using a 7-point scale. The ACQ score is the mean of the 6-items with a theoretical range of zero (well controlled) and six (extremely poorly controlled. The minimum important difference (MID) for ACQ is generally considered to be 0.5 [2]. The MiniAQLQ is an asthma-specific quality of life questionnaire. It consists of 15-items and taps on the problems adults with asthma might find most troublesome in their day-to-day lives in four domains: symptoms, activity limitation, emotional function and environmental stimuli [8]. Patients respond to each question on a 7-point scale from 7= ‘not bothering at all’ to 1 = ‘extremely bothered’. The scores are calculated as the mean of the responses with lower scores suggesting lower quality of life.

The existing Greek versions of the ACQ and the MiniAQOL were used as forwarded by the developers. For the ALMA, the English version, rather than the original Swedish, was used to produce the Greek version using double parallel forward and backward translation (Greek version available from authors at request). The readability of the tool and the need for modifications in terms of the wording or syntax before finalizing, was tested in a pilot study with five asthma patients at the pulmonary clinic of the Nicosia hospital as part their consultation visit.

### Data collection

Eligible participants were identified and informed about the aims of the study by the attending nurse after the examination or during waiting time. Similarly, patients with doctor-diagnosed asthma at the pulmonary ward were informed by the ward staff about the study aim and were invited to participate. Participation was voluntary and anonymous. The questionnaire pack was handed out by the main researcher, who explained the purpose of the study. Participants completed the questionnaires while the researcher was waiting and returned them in a sealed envelope. To facilitate the calculation of the test-retest reliability of the tool, the last three digits of the person’s identity card were noted at the top of the first twenty completed questionnaires in order to allow matching. The second questionnaire was mailed by post to each participant who were requested to return it by post in a sealed envelope noting the last three digits of their ID on the questionnaire.

### Ethical considerations

The study was approved by the Cyprus National Bioethics Committee and the Committee for the Promotion of Research of the Ministry of Health. Furthermore, the Commissioner for the Protection of Personal Data had been notified accordingly. Necessary permissions were also obtained by the administration of the three participating hospitals as well as pulmonary clinic physicians and ward managers. All participants were informed about the aims and objective of the study and they were required to sign an informed consent form. The study did not involve any intervention or interference with normal ward or pulmonary clinic practice. Upon completion, a summary report on the level of asthma control and quality of life on asthma patients was submitted to the pulmonary clinics which participated in the study for their own use.

### Data analysis

Frequencies and relative frequencies of participants’ responses across the 19 items of the ALMA scale were calculated along with the Index of Qualitative Variation. This is a measure of dispersion for categorical variables with a range of 0 to 100% (maximum dispersion observed when an equal proportion of participants is distributed across the response set, i.e. 25% of participants equally distributed across a four-point response scale, or 33% in the case of a three-point response scale). Exploratory factor analysis was used to assess the construct factorial validity of the Greek version of ALMA and compare its dimensionality against the three domains identified in the original Swedish study, namely, physical, psychological and environment triggers. Exploratory factor analysis was appropriate in this case due to the limited validation of the tool in other settings and languages in addition to the unclear structure in the original study (14 vs 17 vs 19 items). The sampling adequacy for factor analysis was assessed using the Keiser-Olkin-Meyer test and the Bartlett test of sphericity. The extraction method applied was principal components. The number of factors to extract were assessed by using the eigenvalues criterion as well as by assessing the scree plot. Since the correlation matrix suggested relatively low correlation between factors, for easy of interpretation varimax rotation was used. Furthermore, the relationship between ALMA and ACQ (often used as the gold standard in clinical and research studies) was assessed as a means of evaluating its convergence validity. Both correlational analysis as well as linear regression were used to estimate the association between one SD increase in ALMA and ACQ. Area under the curve analysis was also performed to calculate the sensitivity and specificity of the ALMA scale in identifying patients with an ACQ score ≥ 1.5, generally considered as predictive of poorly controlled asthma [2]. Concurrent criterion validity had been assessed on the basis of the observed association between ALMA and MiniAQoL. A positive but moderate correlation was expected since self-management and quality of life have been shown to be positively correlated (higher control-better quality of life) but tap on distinct experiences of living with asthma.

The internal consistency of the tool was assessed by estimating Cronbach’s alpha coefficient both for the overall scale as well as the sub-scales of the tool. To assess the stability of the tool, test-retest reliability was assessed among a small sub-sample of twenty participants who completed the questionnaire twice, along with the ACQ in order to assess the stability of a patient’s condition, two weeks apart. Differences in ALMA, ACQ and MiniAQoL by sociodemographic and clinical characteristics were assessed in a series of t-test, ANOVA and Chi-square tests as appropriate. In particular, differences by smoking status and severity of asthma indicators functioned as a test of known-group validity of the measure.

All statistical analysis was performed with the use of SPSS 20. In all cases, the level of statistical significance was set to 0.05.

## Results

The number of participants in the final sample was *N* = 156 who completed all three questionnaires (ALMA, ACQ, mini AQoL) in full. Four participants did not return the questionnaires pack in full, and were excluded from the analysis. Amongst the 156 participants, 95 (60.9%) were women. The median age of the participants was 50–65 years old. As many as 55.7% of the participants were older than 50 years of age, while all other age groups were represented in the sample. As many as 67.3% of the participants had secondary level of education or lower. The relatively low level of educational attainment is not surprising given that more than half the sample was older than 50 years of age. Just over half of the participants (54.1%) were recruited from the larger Nicosia hospital. The rest were recruited from the other two hospitals, specifically 15.9% (*n* = 25) from Larnaca and 29.9% (*n* = 47) from Limassol. The distribution of the sample across the three hospitals roughly represents the population sizes of the three cities.

The socio-demographic and clinical characteristics of participants are presented in Table 1. Just over half of the participants never smoked (54%, *n* = 78); however, 21.7% (*n* = 34) used to smoke and 28.2% (*n* = 44) are current smokers. Current smokers (*N* = 44) have reported smoking on average 21 (SD 12.3) cigarettes/day over an average period of 18 years (SD 11.4). Past smokers (*N* = 34) reported smoking on average 25 (SD 18.6) cigarettes/day when they used to smoke, and they smoked on average for 15.4 years (SD 12.5). In total, among current and past smokers (*N* = 78), the mean pack-years of smoking was estimated at 488 (SD 644) (equivalent, say to a pack of 20 cigarettes a day for a period of 20 years) and a range of 15 pack-years (say, equivalent to 5 cigarettes a day for 10 years) to 4000 pack-years (say, equivalent to three packs a day for 66 years) - results not shown in detail.

**Table 1:** Socio-demographic and clinical characteristics of the participants

Variable		Frequency (*N* = 156)	Relative Frequency (%)
Gender	Male	61	39.1%
	Female	95	60.9%
Age	18–30	29	18.5%
	30–40	20	12.8%
	40–50	20	12.8%
	50–65	57	36.5%
	65 and over	30	19.2%
Education	Primary	39	25.0%
	Secondary	66	42.3%
	Undergraduate	34	21.8%
	Postgraduate	17	10.9%
Smoking status	Current	44	28.2%
	Past	34	21.7%
	Never	78	54.0%
Family history of atopy	Yes	62	39.4%
	No	94	60.6%
Drug allergy	Yes	27	17.4%
	No	129	82.6%
Comorbidity status	Yes	76	48.7%
	No	80	51.3%
Comorbidity condition	Gastro. reflux	15	19.7%
	Sinusitis	20	26.3%
	Nasal polyps	6	7.9%
	Rhinitis	15	19.7%
	Res. Infection	15	19.7%
	COPD	5	6.6%
District	Nicosia	85	54.1%
	Larnaka	25	15.9%
	Limassol	47	29.9%

There was a wide variability in the ALMA scores across participants. The distribution of overall scores did not deviate from the normal distribution (Kolmogorov-Smirnov test *p*-value = 0.2) with a mean of 38.3 (SD 10.3). The range of observed scores was 18–62 (theoretical range 17–67) and the interquartile range (IQR) was 31–45. Table 2 shows the distribution of responses per individual item of the ALMA. In general, it appears that the most frequent positive responses were recorded in the items which refer to environmental conditions (smoke, dust, cold) for which more than half of the participants responded that these affect them “often”. As a result of the ceiling effect observed in the case of the environmental domain items, a lower dispersion of responses was observed across the four-point response scale as indicated by the Index of Qualitative Variation. Relative high frequency was also observed in the case of some of the mental domain items. For example, as many as 55.4% of the participants responded that they “often think and worry about their asthma”. However, other than these exceptions, there appeared to be relatively high variability across the four-point response set in all other items as indicated by the IQV. The least frequent negative responses were recorded for item “I have tightness in the chest” for which only 1.3% responded often and 17.2% responded sometimes. The remaining 81.5% responded rarely or never. Nevertheless, in terms of using medication for rapid relief, 37.6% of the participants responded that they do so more than two times a week and 28.0% twice a week. One in three (34.4%) stated that they never used any medication. In terms of emergency visits or hospitalization in the past year, 31.2 and 20.4% responded positively.

**Table 2:** Participants’ responses on the Active Living with Asthma questionnaire and Index of Qualitative Variation

	Often	Sometimes	Rarely	Never	IQV
Item 1: Tightness in the chest	8.9%	45.2%	36.9%	8.9%	85.7%
Item 2: Severe cough even when not have a cold	19.1%	34.4%	28.7%	17.8%	97.5%
Item 3: Dust, pollen and animal fur make asthma worse	51.6%	26.8%	10.8%	10.8%	85.1%
Item 4: When cold, asthma worsens/ difficult to breath	27.4%	33.8%	22.9%	15.9%	97.7%
Item 5: Difficult to breathe when exposed to cigarette smoke and strong odours	54.8%	19.7%	17.2%	8.3%	83.2%
Item 6: When get a cold, asthma worsens/ difficult to breath	54.1%	29.9%	12.1%	3.2%	79.6%
Item 7: Think about asthma and worry	55.4%	22.3%	12.1%	10.2%	82.4%
Item 8: Asthma affects life more than would want	22.3%	33.1%	28.7%	15.3%	97.6%
Item 9: Do not do as many things as would like	20.4%	36.9%	22.9%	19.7%	97.4%
Item 10: Cough/ difficulty breathing when walking or tired	35.7%	30.6%	21.0%	12.7%	95.8%
Item 11: Cough/difficulty breathing when heavy or intense work	35.0%	29.9%	22.3%	12.7%	96.2%
Item 12: Cough/ difficulty breathing when participating in sports activities	30.6%	33.1%	16.6%	19.7%	97.4%
Item 13: Wake up with cough and difficulty breathing	12.1%	34.4%	25.5%	28.0%	96.5%
Item 14: Wheeze when breathing	18.5%	37.6%	31.2%	12.7%	94.8%
Item 15: Asthma symptoms despite taking medications	24.2%	36.9%	26.1%	12.7%	96.1%
Item 16: Medications cause discomfort	1.3%	17.2%	31.2%	50.3%	82.7%
	**Yes**	**No**			
Item 17: Emergency department visit past year	31.2%	68.8%			85.9%
Item 18: Hospital admission in past year	20.4%	79.6%			65.0%
	**Never**	**Up to twice/ week**	**More than two times/ week**		
Item 19: Rapid-relief medications	34.4%	28.0%	37.6%		99.3%

The KMO = 0.83 and Bartlett test < 0.001 suggested acceptable sampling adequacy to proceed with factor extraction. Exploratory factor analysis of the 19 items of the scale with varimax rotation initially revealed four factors. Factor 4 loaded on two items which are the two binary (YES/NO) items referring to hospital admissions or emergency department visits in the last year. When the analysis was repeated without these two items, also excluded from the original study, a clear structure of three factors with minimal cross-loading was observed roughly along the lines of the three domains as per the original study: physical, environmental, mental factors, explaining 54.6% of the total variance. The rotated factor loadings pattern is shown in Table 3 where only factor loadings with a value of > 0.39 are shown. It is also worth noting that item 13 (referring to the use of rapid relief medication) loads negatively as expected because this item is reverse coded.

**Table 3:** Exploratory factor analysis of the 17-item Active Living with Asthma scale

	Factor 1 (Physical)	Factor 2 (Environmental)	Factor 3 (Mental)
…. when walking	0.86		
… heavy or intense work	0.84		
… participating in sports activities	0.74		
…night time awaking	0.73		
…wheezing	0.55		0.49
…severe cough/ not cold	0.54		
…taking medication	0.50		
…chest tightness	0.41		
…dust, pollen, animal fur		0.77	
… cigarette smoke and strong odours		0.74	
…cold outdoors		0.69	
…a cold		0.60	
…rapid relief medication			−0.77
…worried			0.70
…affect daily life			0.64
…refrain from things	0.46		0.55
…medicine discomfort	0.44		0.45
Cronbach’s a	0.85	0.69	0.76

The original study retained only 14 items, as three items did not load on any of the factors. In the case of this study, it was decided to retain these three items, namely “I have asthma symptoms despite the fact that I take my medications as prescribed by my doctor” in factor 1 as well as “My medications cause discomfort” and “I use rapid relief medications in factor 3”. Thus, in this study the items were distributed across the three scales as follows: physical factor– 8 items (all same as original study plus one), mental factor – 5 items (3 original plus two) and environmental factor – 4 items (all same as original). Cronbach’s alpha coefficient for internal consistency for the whole scale was 0.85 while for the sub-scales, these were: factor 1 - physical factor a = 0.85 (8 items), factor 2 – environmental factors a = 0.69 (4 items) and in factor 3 – mental factor a = 0.76 (5 items). Finally, test-retest reliability was assessed using Pearson’s correlation coefficient at r = 0.92.

The convergence validity of the ALMA was evaluated against the 6-item ACQ scores (Cronbach’s alpha coefficient estimated at 0.89 and test-retest reliability estimated at r = 0.96. The mean ACQ score was 1.5 (SD 1.27) with an observed range of 0–4.8. It should be noted that while higher scores indicate better levels of self-management in the case of ALMA, in the case of ACQ higher scores indicate worse asthma control. As many as 49.3% of participants in this study had an ACQ score ≥ 1.5, commonly, taken to be predictive of poorly controlled asthma. Area under the curve analysis suggests that a score of 35.5 on the overall ALMA scale has 83.0% sensitivity and 69.6% specificity to identify patients with an ACQ score ≥ 1.5. The correlation between ALMA and ACQ was − 0.70. High correlations were observed between the ACQ and all sub-scales of the ALMA with the higher correlation observed with the physical domain (r = − 0.68) as expected since the ACQ is focusing exclusively on physical symptoms. A clear stepwise pattern of decreased ALMA scores, indicating worse self-management, was observed across quartiles of participants with higher ACQ scores (indicating worse asthma control). The differences were apparent for the overall score and the three sub-scale scores, and were in all cases statistically significant (*p* < 0.001) – see Table 4. Furthermore, a standard deviation increase in the overall ALMA score is associated with a − 0.86 (95% CI -1.00, − 0.72) difference in the ACQ score as estimated in a linear regression model (not shown in detail); hence, 1 SD difference in the ALMA score appears to reflect a clinically significant difference given that the minimum important difference for ACQ is generally considered to be 0.5 [2].

**Table 4:** Differences in ALMA scores according to ACQ as well as socio-demographic and clinical characteristics

	Overall Mean (SD)	Physical Mean (SD)	Mental Mean (SD)	Environmental Mean (SD)
Theoretical range	17–67	9–27	4–15	4–16
All participants	38.3 (10.3)	22.1 (6.0)	8.6 (3.0)	7.6 (3.6)
Emergency visits				
Yes	33.7 (8.4)	19.8 (5.3)	7.3 (2.7)	6.6 (2.3)
No	40.3 (10.4)	23.1 (6.1)	9.1 (2.9)	8.1 (4.0)
p-value	< 0.001	0.001	< 0.001	0.11
Hospital admissions				
Yes	32.8 (7.7)	19.6(4.9)	6.8(2.7)	6.2(1.8)
No	39.7 (10.4)	22.7(6.1)	9.0(2.9)	8.0(3.8)
p-value	0.001	0.11	0.001	0.10
Age				
18–29	46.2(9.0)	25.4(5.8)	11.2(2.0)	9.5(3.2)
30–39	38.4(12.0)	21.3(6.3)	9.6(3.1)	7.5(4.0)
40–49	33.4(8.2)	19.3(4.5)	7.2(2.6)	6.8(2.6)
50–64	36.4(8.6)	21.7(5.7)	8.1(2.6)	6.5(2.4)
65 and over	37.5(10.9)	22.0(6.3)	7.0(2.8)	8.5(5.0)
p-value	< 0.001	0.006	< 0.001	0.003
Education				
Primary	39.3(10.8)	23.5(6.7)	7.9(2.6)	7.9(2.9)
Secondary	35.4(10.0)	20.2(5.7)	7.9(2.9)	7.3(4.0)
Undergraduate	40.6(9.0)	23.7(5.0)	9.5(3.0)	7.4(3.3)
Postgraduate	42.5(10.2)	23.2(5.7)	10.6(2.8)	8.7(3.3)
p-value	0.16	0.008	0.001	0.449
Smoking				
Current	43.0(11.2)	22.8(6.6)	10.0(3.3)	10.2(4.3)
Past	36.9(10.5)	21.8(6.2)	8.0(2.8)	7.2(3.2)
Never	36.2(8.8)	21.8(5.5)	8.0(2.5)	6.3(2.4)
p-value	< 0.001	0.368	< 0.001	< 0.001
ACQ scores quartiles				
Q1: < 0.5	48.1 (7.9)	27.8 (4.4)	10.6 (2.5)	9.6 (3.5)
Q2: 0.5–1.2	38.8 (8.0)	21.3 (4.2)	8.8 (2.8)	8.5 (4.7)
Q3: 1.2–2.5	35.3 (5.7)	21.3 (3.9)	7.7 (2.1)	6.2 (2.1)
Q4: > 2.5	29.3 (7.5)	16.7 (4.6)	6.7 (2.7)	5.8 (2.0)
p-value	< 0.001	< 0.001	< 0.001	< 0.001

The correlation between AQLQ and ALMA was 0.71. High correlations were observed between the AQLQ and ALMA domains, in ranking order: physical r = 0.62, r = mental 0.59 and environmental r = 0.51. It also important to note that that there was a good level of consistency between the various domains of asthma control/self-management and the corresponding aspects of quality of life as indicated by the higher correlations observed between matching domains. For example, the physical domain of ALMA showed higher correlations (in the magnitude of 0.6) with the symptoms and activities aspect of quality of life, compared to the other aspects of quality of life (0.3–0.5). Similarly, the environmental domain of ALMA showed higher correlation with environmental-related quality of life (in the magnitude of 0.6) compared to the other aspects of quality of life (in the magnitude of 0.4) – results not shown in detail.

Table 4 also shows the observed differences in the overall score as well as the three sub-scale scores according to socio-demographic and clinical characteristics. In terms of age, generally better scores in all domains were observed among the younger and older age-groups and worse scores in the middle age groups. Higher scores at least for the physical and mental domains (*p* < 0.001) but not for the environmental domain were observed among people with higher educational attainment. In terms of smoking, worse scores were observed among current smokers for the environmental domain (*p* < 0.001), mental domain (*p* < 0.001) and overall score, but not for the physical domain. Finally, statistically significant differences were observed between participants who reported emergency visits and hospital admissions in the past year. While these differences appeared larger with regards to the overall score, physical and mental score, differences in environmental domain were in the same direction, even if not always statistically significant at the 5% level. No statistically significant differences were observed in terms of gender, family history of atopy, drug allergies or district of sampling.

## Discussion

### Main findings

The ALMA showed good metric properties amongst a sample of 156 adult asthmatics in Cyprus. Factor analysis revealed the postulated dimensionality of physical, environmental and mental domains of asthma self-management, with good internal consistency and test-retest reliability in the measurement. With a few exceptions pertaining mainly to the environmental domain, there were no indications of floor or ceiling effect in any of the items. Furthermore, the ALMA showed good convergence validity with the shorter ACQ as well as good concurrent criterion validity in terms of the observed association between the various domains of asthma self-management and the corresponding aspects of quality of life. Finally, the known-group validity of the scale is supported by the lower observed ALMA scores among smokers as well as according to emergency visits and hospital admissions in the past year as indicators of asthma severity.

### Strength and limitations

This is the first study to translate and evaluate the metric properties of an asthma review questionnaire among Greek-Cypriot asthmatic patients. In fact, we are not aware of any study that used the ALMA beyond the original setting (Sweden) where it was developed. The participants were selected from pulmonary clinics across the three largest public hospitals in Cyprus. Due to the study design (three specialised centres) and sampling (consecutive voluntary sample), the findings in terms of the level of asthma control and experience of asthma patients in terms of management of their symptoms are not generalizable to other settings or even all asthma patients. While convenience sampling in three specialised centres was used, the prime purpose of this methodological study was the evaluation of the metric properties of the tool and not the depiction of the level of asthma control among asthma patients in Cyprus. The sample was heterogeneous in terms of their socio-demographic (age, education etc) and clinical characteristics (family history, comorbidity etc). In the absence of official statistics on the socio-demographic characteristics of asthma patients in public hospitals in Cyprus, it is not possible to assess the representativeness of the sample. In terms of gender, while there is evidence to suggest that in several countries both the prevalence as well as hospitalization for asthma in more common in adult women, there are no similar statistics from Cyprus. The female-to-male ratio observed in this study is lower than the 2:1 commonly observed elsewhere. It is unclear whether this reflects the gender distribution in Cyprus or it suggests more willingness among men to participate in the study. Finally, the convergence validity of ALMA was assessed against the “gold standard” ACQ questionnaire. For convenience purposes, the last item of the ACQ was not included since it refers to prebronchodilator forced expiratory volume in one second (FEV1). Thus, other than self-reported measures, the study did not have any objective measures of asthma control. Nevertheless, self-reported service utilization measures were collected, as they form part of the ALMA questionnaire (admissions to hospital and emergency department visits). Thus, the study was able to show differences in asthma control according to these measures of asthma severity, which were in the expected direction.

### Structured asthma reviews

Self-management education is considered to be a vital aspect of asthma care as patients need to be educated to recognise and self-manage their symptoms and thus reduce the risk of life-threatening exacerbations and long-term morbidity [9]. The British Thoracic Society/Scottish Intercollegiate Guideline Network (BTS/SIGN) asthma guideline cites 261 randomized controlled trials reported in 22 systematic reviews in support of its grade A recommendation that ‘all people with asthma should be offered self-management education which should include a written personalized asthma action plan and be supported by regular professional review [3]. Nevertheless, studies have suggested that both patients and physicians alike overestimate the level of asthma control [10]. There have not been any studies to date among Cypriots adult asthmatics. Anecdotal evidence suggests that many patients perceive their asthma to be mild and well-controlled despite reporting frequent symptoms. Also, studies suggests that patients with asthma and other chronic diseases do not perceive that working out a preventative strategy is worth the time and effort involved. [11]. The extent to which asthmatics in Cyprus have low adherence to treatment guidelines and poor knowledge of the disease is not known. While education and written personal management plans may be used in clinical practise in Cyprus, this is certainly not routine practice. Information and advice for asthma is available through leaflets and subscribed from specialist doctors during or after their examination. The effectiveness of patient education and asthma management plans have not been systematically assessed. The lack of a standardized audit and review tool in the Greek language may contribute further to the lack of a standard practice as well as research in this field.

Several instruments have been developed for measuring asthma control. In their review, Alzahrami and Becker [1] identified: the Asthma Control Test (ACT), the Asthma Control Questionnaire (ACQ), the Asthma Therapy Assessment Questionnaire (ATAQ), the Lara Asthma Symptom Scale (LASS) and the Childhood Asthma Control Ttest (CACT). The authors also identify a number of limitations for each tool. Whereas the ACQ and ACT are closely aligned with the 2015 GINA [6] and NAEPP EPR-3 guidelines, neither tool assess the risk of asthma exacerbation, which is an integral part of both guidelines criteria of asthma control. Despite certain limitations, the ACQ has been extensively used internationally and often considered the gold standard, especially as a primary or secondary endpoint in clinical trials [2]. However, the ACQ as well as other available and validated tools focus exclusively on the control of physical symptoms, whereas the ALMA taps on wider aspects of asthma control, including asthma triggers and self-management and can, thus, form the basis of a structured review in primary care in the context of patient education and personalized asthma action plans. A recent Cochrane review concluded that there was no strong evidence that there was either benefit or harm of personalized asthma action plans, either compared to no action plans or as an additional component to patient education, in the management of adult asthma [5]. However, among fifteen Randomized Control Trials reviewed, the endpoint was commonly emergency admissions and hospitalizations following exacerbations and/or lung function. Only two studies used level of asthma control (using the ACQ) and four studies used quality of life (using the AQLQ) as an endpoint, with the latter reporting a statistically significant improvement but below the Minimum Clinically Important Difference. The authors concluded that further research is required, to quote, “with a particular focus on other key patient-relevant outcomes”. Since the ALMA covers a wider spectrum of asthma control and self-management, it could provide an additional Patient Reported Outcome Measure (PROM) in such future studies.

### Patient reported measures of asthma control and self-management

As accurately described by Pinnock et al. [15], beyond the validation process, the choice of PROMs (Patient Reported Outcome Measures) has to reflect the specific purpose of their intended use as well as the setting. For instance, the Royal College of Physicians RCP3Q comprises of only three questions related to asthma control, namely night symptoms, day symptoms and interference with usual activities. It has been assessed against the ACQ and predicted poorly control asthma with high sensitivity (0.96 but low specificity (0.34). Nevertheless, as it is easy to implement in clinical practice, it is the measure of choice by the UK’s Quality and Outcome Framework for reviewing standards of clinical practice in asthma care.

A core function of an asthma review is to assess control as well as to provide an assessment of the impact on the patient’s life and improve self-management of the symptoms. Hence, a measure which may be more appropriate to use as an endpoint in a research study is not necessarily fit for use in clinical practice if the main purpose is patient education. Evaluation of the ALMA tool suggests that the items cover key topics in an auditable structure for primary care asthma reviews (i.e. physical restrictions, environmental, mental and healthcare utilisation). These are important aspects when assessing patients with asthma, and the use of the ALMA tool may help structure this evaluation as well as function as a patient self-management education tool. As we have shown, the ALMA has a good correlation with an established instrument of asthma control (ACQ) while at the same time the inclusion of a mental and environmental domain of self-management in the ALMA tool allows for an integrated approach in patient education and self-management review.

A study of over 1200 asthma patients across five European countries found that over 50% report exposure to 6–15 asthma triggers [17]. Environmental triggers, such as dust, tobacco smoke, animal fur, etc., ranked amongst the triggers most likely to be experienced by participants (reported by at least 50%) as well as the triggers that are more likely to be experienced often. Patients with a high trigger burden were more likely to report uncontrolled asthma and have more severe attacks, more hospitalizations, higher number of missed days from work and more avoidance or behaviour changes to manage trigger exposure.

In this study, more than half of the participants responded that they have asthma aggravations “often” in three out of four environmental domain items. It is interesting to note that these were also the items with the highest observed frequency of positive responses among Swedish asthma patients, which nevertheless was restricted to around one in three responding positively to being frequently exposed to “dust, pollen, animal fur” or “tobacco smoke or strong odours”. [10]. The higher frequency observed in Cyprus is not thought to be a ceiling effect but to reflect the actual high prevalence of these environmental exposures. A secondary analysis of Eurobarometer data indicated that Cyprus ranks in the top positions in terms of exposure secondhand smoke (SHS) among 27 EU countries, surpassed only by Greece, Bulgaria and Romania, with over 70, 50 and 35% of non-smokers reporting SHS in bars, restaurants and in the workplace respectively [4].

Validation of a tool is an iterative process and further studies should explore the responsiveness and interpretability of the ALMA scores. While the scale appears to be a valid measure to use in clinical settings to assess self-management of asthma symptoms, the study has not explicitly assessed the interpretability of the ALMA scores. There is some indication to suggest that a SD increase in the overall ALMA score is associated with a minimum clinically important difference in asthma control, as assessed by the ACQ. However, further studies need to be performed in order to assess the MID of ALMA against self-reported and objective measures of asthma control. Furthermore, the responsiveness to change in response to patient education or other healthcare interventions should be assessed in longitudinal experimental or quasi-experimental studies. Furthermore, beyond the validity and usefulness of the measure, the introduction of a PROM in real clinical settings involves an assessment of additional aspects, such as the acceptability by the healthcare providers, the administrative burden for the clinicians as well as the various possible modes and methods of administration [13].

## Conclusion

This study provided some first-time information about the level of self-management among Cypriot asthma patients. More importantly, the study assessed the metric properties of an asthma self-management tool in a new setting and specifically the validity (construct, criterion, convergence and known-group) and reliability (internal consistency and test-retest). The ALMA showed good metric properties and could be used as measure for asthma control and self-management in future descriptive or intervention research studies. Primarily, it can be used in clinical practice as a patient education tool and as a structured review tool in the context of the systematic assessment and monitoring of asthma control and self-management.

## Abbreviations

ACD: Asthma Control Diary
ACQ: Asthma Control Questionnaire
ALMA tool: Active Life with Asthma
HRQoL: Health related quality of life
MiniAQoLQ: Mini Asthma quality of life questionnaire
